# Early Childhood Caries and the Impact of Current U.S. Medicaid Program: An Overview

**DOI:** 10.1155/2012/348237

**Published:** 2012-03-08

**Authors:** Bussma Ahmed Bugis

**Affiliations:** ^1^Division of Management, Policy and Community Health, School of Public Health, University of Texas Health Science Center at Houston, Houston, TX 77030, USA; ^2^Saudi Arabian Cultural Mission to the U.S., Ministry of Higher Education in Saudi Arabia, Riyadh 11153, Saudi Arabia

## Abstract

Pediatric dental caries is the most common chronic disease among children. Above 40% of the U.S. children aged 2–11 years have dental caries; more than 50% of them come from low-income families. Under dental services of the Medicaid program, children enrolled in Medicaid must receive preventive dental services. However, only 1/5 of them utilize preventive dental services. The purpose of this overview is to measure the impact of Medicaid dental benefits on reducing oral health disparities among Medicaid-eligible children. This paper explains the importance of preventive dental care, children at high risk of dental caries, Medicaid dental benefits, utilization of dental preventive services by Medicaid-eligible children, dental utilization influencing factors, and outcome evaluation of Medicaid in preventing dental caries among children. In conclusion, despite the recent increase of children enrolled in Medicaid, utilizing preventive dental care is still a real challenge that faces Medicaid.

## 1. Introduction

Dental caries is found to be the most common chronic disease among children [1]. Oral health disparities among children still exist in the United States. According to *Oral Health in America: A report of the Surgeon General, *children from minorities who come from low-income families are at higher risk of having untreated dental caries and poorer access to dental services than their peers from majority and high-income families [2]. In general, above 40% of the U.S. children aged 2–11 years have dental caries, and more than 50% of them come from families with income less than 200% of the federal poverty level [3]. When predicting the development of oral diseases, history of having dental caries is the most predicting factor of future caries [4]. Invasive dental treatments can be reduced among children at high risk with proper preventive dental care (PDC). Savage and colleagues [5] found that preschool-aged children receive the highest level of PDC among other ages and the early they receive PDC, the more likely they utilize PDC regularly and the fewer costs related to dental treatments.

 Receiving preventive dental services is mandated benefit for children enrolled in Medicaid as a part of the early and periodic screening, diagnostic and treatment (EPSDT) benefit under dental services of Title XIX of the Social Security Act, the Medicaid program [6]. However, not all Medicaid-insured children have an equal access to PDC. In fact, only 1 child out of 5 Medicaid-eligible children receives PDC, as reported by the Office of Inspector General [7]. Although Medicaid provides comprehensive dental benefits for eligible children and prohibits copayments for preventive care, multiple barriers affect the utilization of PDC both within dental delivery services and Medicaid-eligible families.

 The purpose of this paper is to measure the impact of Medicaid dental benefits in increasing the awareness of preventive dental services and decreasing oral health disparities among Medicaid-eligible children. In addition, the utilization of PDC along with influencing factors will be explained briefly. Outcome evaluation of Medicaid in eliminating oral health disparities among the U.S. children will also be assessed. Finally, recommendations and suggestions will be provided.

 The areas to be covered are the following: first, the importance of early childhood preventive dentistry; second, children at high risk of dental caries in the U.S.; third, dental benefits under Medicaid; fourth, accessing and utilizing dental preventive services by Medicaid-eligible children; fifth, contributing factors of preventive dental utilization at micro, meso, and macro levels; sixth, outcome evaluation of Medicaid impact in preventing dental problems among children; lastly, conclusion.

## 2. Early Childhood Preventive Dentistry

The first area to be covered is the importance of obtaining early PDC. Children with dental problems such as dental caries or poor oral hygiene face a lot of issues related to their oral health and overall health status. In the U.S., it is estimated that 2% of infants 12 to 23 months of age have at least one tooth with questionable tooth decay and 19% of children 24 to 60 months of age fit into early childhood decay definition of having dental decay in the first 5 years of age or younger [8]. The only U.S. population that has experienced an increase in the level of dental caries is young children [9]. In fact, only 23% of children 8 years or younger had received dental sealants [2] which are placed mainly in the pits and fissures of the occlusal surfaces of individual molars to prevent future dental caries [10].

 Children with dental caries usually gain weight much slower than their peers without dental caries. Dental caries can prevent the child from eating and getting proper nutrition [11]. It is significantly approved that kids with early dental caries are more likely to weigh less than 80% of their ideal body weight indicating that having early dental caries may affect a child's growth in a negative way [12]. Children with early childhood caries (ECC) not only their oral health and overall health are negatively affected, but also different ramifications. For example, with emergency dental visits, a child might miss hours in attending school or learning activities, plus parents may have to excuse from their works. In addition, pain relief visits or emergency dental visits would make a bad impressions in the child's mind that dental office is only related to pain. Furthermore, the more severity of oral health status, the more necessity of complex dental treatments which are more expensive than preventive or regular dental visits. Other negative effects of ECC are speech problems, low self-esteem, and low proper concentrate [11].

 Dental caries in children are 100% preventable, and future dental problems might be reduced or eliminated with early proper prevention strategies. Preventive dentistry for kids is identified as “dental care designed to maintain healthy teeth and prevent problems” [11]. The American Academy of Pediatrics recommends that a child should receive a first dental risk assessment at 6 months of age and start dental home practice by the first year of age for children at high risk of dental caries [13]. No later than 12 months of age, infants are recommended to see dental professional as their first dental visit [14].

 Preventive dental care is important in educating the child and the parents, identifying current dental status, and preventing future dental problems. Dental sealants showed to be effective in reducing the possibility of having dental restorations in sealed molars up to 7 years after placing sealants for children at high risk of oral cavities [15]. In addition to placing sealants, the use of fluoride reduces dental caries, and it can be delivered through water, toothpaste, rinses, and professional applications [16]. Regarding costs, children who had received early PDC usually have lower dental-related costs than children who started preventive strategies at a later stage [5].

## 3. Children at High Risk

The second discussion is the identification of children groups who are at high risk of dental problems. Over the last ten year, oral health problems are known to be related to children at different ages. More than 30% of children who live below the poverty level do not receive treatments for their dental caries compared to only 6% of their peers who live just at 300% or higher of poverty level [8]. Many studies have shown that young children from low-income families and minority groups are at high risk of having oral health problems [3, 17, 18]. There are many reasons that explain the increasing prevalence of dental problems among children with special needs and who come from low socioeconomic status and minority groups.

 Vargas and Ronzio wrote a literature review on childhood dental caries disparities published in 2006 [19], and they found that children who are socially disadvantaged of being race/ethnic, minority, low SES, immigrants, and live in rural areas, have experienced an increase in the prevalence of oral diseases. Authors indicated that low-income children are in most need for dental treatments and least likely to be seen by dentists for oral care. Reasons for not seeking dental care include limited resources with different priorities and difficulties in finding a dentist who accept uninsured or publically insured children. They concluded that interventions to eliminate oral disparities health among children have not reached all low-income children 2–5 years in the community [19]. 

 A following paper on oral health disparities published in 2009 agreed with Vargas and Ronzio [19] that minority, young, poor, and low-income children have experienced greater number of decayed teeth than higher income majority children [3]. More than 42% of the total U.S. children population has at least one tooth decay, and more than 25% of 2–6 years were infected. Authors indicated that disparities in receiving dental care still exist between children from high-income families and their peers from low-income families. They found that 40% of low-income children have received dental treatments compared to 54% of higher-income children [3].

 Furthermore, a longitudinal study was conducted among young children of low-socioeconomic status (SES) to predict factors that are associated with dental caries besides being from low SES [17]. One hundred and twenty-eight children were followed for 18 months from a rural community in Iowa. They found that sociodemographic and fluoride consumptions are statistically not correlated with dental caries prevalence. Importantly, they suggested that the consumption of sugar-sweetened drinks during the first two years of birth and the presence of mutans streptococci are the strongest predictors of dental caries among low SES children [17].

 Children aged 6–10 years do not differ much from very young children in regard to dental caries and oral health problems. A cross-sectional analytical study of the 2007 National Survey of Children's Health was conducted [18]. Researchers explained that known states of high percentage of population living in or below poverty level such as Mississippi have more children with dental caries. On the other hand, toothache negatively correlated with per capita dentist supply as it is the case in the Districts of Columbia and Massachusetts. In general, the U.S. children at 200% or low of FPL, minority, and those with special needs are significantly at high risk of having oral health problems [18].

 In addition to SES, different racial and ethnic groups are at high risk of untreated dental decay. For the last ten years, children who are African-Americans and Mexican-Americans have experienced more dental caries than White-Americans [20]. Unfortunately, the gap to decrease oral health disparities between American Indian/Alaska Native populations is huge comparing to the rest of the U.S. populations. American Indian/Alaska Native children aged 2–4 years are five times more likely to have oral problems compared to other children [20].

## 4. Medicaid

Medicaid dental coverage and its impact on PDC are going to be briefly discussed in this section. Under Title XIX of the Social Security Act, the federal Medicaid statute was enacted in 1965 [21]. Medicaid is a kind of federal financial participation that is “a means-tested, social welfare program that uses tax revenues to provide health coverage for persons who cannot afford private insurance” [22]. Eligibility for Medicaid is restricted to individuals who are U.S. citizens or “qualified aliens” immigrants with limited income and assets, and others who are specially categorized in need for public assistance such as people with very high medical expenses that exhaust most of their resources [22].

 All Medicaid beneficiaries are eligible to receive unlimited coverage on some particular types of illness; however, their eligibility to receive other certain health services is limited and depends on medical necessity [22]. Individuals who are under age 21 are required to receive EPSDT to be eligible for Medicaid benefits [22]. Having regular dental care services is mediated as part of EPSDT for those under the age of 21 years [6].

 Not only dental emergencies are covered under Medicaid, but also reasonable dental benefits which include at least infection and pain relief, build-ups and restoration of teeth infected with dental caries, and oral health maintenance [6]. Oral screening administered during physical exam is not enough. A referral to a dentist followed with dental examination performed by a dentist is required. Medicaid dental services work through intervals at both dental organization intervals to perform oral consultation and medical providers to determine medical necessity. All expenses are covered as long as the term of medical necessity is fulfilled [6].

 Medicaid funding comes from both federal and state government which makes both governmental levels operate the state Medicaid services [22]. States are required by federal law to provide Medicaid-eligible children, until age of 21 year, with access to EPSDT and effectively utilize available resources [7].

 The Office of Inspector General from the Department of Health and Human Services listed requirements for states to help improving access and utilization of dental services covered under Medicaid [7]. The requirements for states enrolled in Medicaid, provided by the Office of Inspector General are the following.

1. Medical and dental providers including physicians, dentists, and others should be recruited to participate in EPSDT.
2. Medical and dental examinations, diagnosis, and treatments should be monitored and overlooked.
3. Families who are Medicaid-eligible should be located and informed about EPSDT.
4. The desired services of medical, dental, vision, and hearing screenings should be fit into schedules based on the practice of professional standards.
5. The Health Care Financing Administration (HCFA) should be provided with fully informed report on the utilization of EPSDT services.
6. Any condition requires treatment identified by a screening and needed services must be provided, whether or not this service is included in the state's Medicaid plan [7].

As any nonprofit task, Medicaid faces a challenge of the rapid increase in expenses with very limited budgetary. This challenge affects the quality and the access to care. Many states find the best way to reduce costs is through reducing the amount of reimbursements to very low levels to satisfy the federal statute of sufficient payment [22]. This action can highly influence the decision, as health services provider, to participate in Medicaid.

 Studies have shown that Medicaid low reimbursements is negatively correlated with the number of dentists participated in Medicaid. In Alabama, a survey was mailed in 2003 to 518 Medicaid dentists had a response rate of 54% aimed to examine the relationship between Medicaid dentists and their participation in Medicaid [23]. Researchers included in their conclusion that dentist' perceptions of Medicaid, such as high reimbursements and quick process of receiving payments have a high influence on their continuing participation [23].

 A similar study conducted in Louisiana found that Medicaid reimbursements is much less than private payments [24]. Louisiana dentists were not satisfied with Medicaid due to low fees, slow mechanism of receiving payments, unreasonable denial of payments, and complications in filling paperwork for Medicaid [24].

 Not surprisingly, Connecticut dentists are unwilling to accept new Medicaid children into their practices mainly because of the low reimbursements rates for children [25]. In North Carolina, the case is not much different than other states. It has been documented that only 16% of dentists in NC participate in the Medicaid policy [25].

## 5. Access and Utilization of Dental Preventive Services

The fourth section of this paper presents the access and the utilization of PDC among Medicaid-eligible children. People are confused and cannot differentiate between access and utilization of dental care. Measuring access and utilization is not an easy task. In fact, within access and utilization of dental care, there are many subvariables that are critical when measured. The definition of access to dental care is “the ability to gain available, appropriate services as determined by personal, economic, cultural, geographic, and other factors” [26]. On the other hand, utilization of dental care is related to patients' or families' familiarity and their uses of available dental resources effectively [27]. Having an accessible dental care does not necessarily mean utilizing it; however, utilizing dental care means accessibility is approached.

 Many researchers have addressed the issue of access and utilization of PDC among Medicaid-enrolled children. A longitudinal retrospective cohort study found that 23% of 15,438 Medicaid-enrolled children receive at least a single dental caries prevention [15]. Other study did not find the percentage of 31% children enrolled in Medicaid who received dental services convincing, leaving about 69% without any kind of dental services in Maryland [27].

 A North Carolinian 5-year cohort study found that preschool-aged children enrolled in Medicaid who have a history of at least one preventive dental visit (PDV) are more likely they utilize PDC in the future and lower dental-related costs [5]. Researchers found that the utilization of PDC is positively correlated with the number of available dentists and previous PDV but negatively correlated with restorative dental visits if PDC was received before the age of one year. They also included that minority of Medicaid-enrolled children are less likely to utilize dental services in general [5].

 Many studies from different states such as Connecticut [28], Michigan [29], Alabama, and Georgia [30] share North Carolina the same result that Medicaid-enrolled children who utilize PDC receive less dental treatments, caries restorations, and comprehensive dental care than non-Medicaid children [31]. Furthermore, Washington State found that the majority of dental procedures performed on Medicaid-enrolled children among 3,244 different dental procedures are preventive and diagnostic procedures [32]. 

 The type of dental provider also affects the utilization of PDC. Medicaid-enrolled children in New Hampshire are more likely to receive comprehensive PDC when they are seen by pediatric dentists than those who receive PDC from general dentists [33]. Pediatricians and general physicians are major players in referring Medicaid-eligible children to dentists for PDC. It has been suggested that it is difficult for pediatricians to comply with EPSDT if each and every single community does not have enough dentists who accept Medicaid beneficiaries [34].

 Children identified as children with special health care need (CSHCN) who are Medicaid-enrolled children are also eligible for full dental coverage. This special group of children, aged 1–17 years, do not receive PDC as much as CSHCS who are not enrolled in Medicaid because of the low reimbursements of Medicaid dental procedures [35]. Another comparative preventive dental utilization study was made in Iowa by Chi and colleagues [36]. Investigators from Iowa compared intellectual and/or development disability (IDD) Medicaid-enrolled children with peers without IDD to study their utilization of PDC. They concluded that regardless of the barriers that may face IDD children, both Medicaid-enrolled IDD children and those without IDD utilize PDC just as the same level [36].

 According to the Office of Inspector General, there are fewer Medicaid-eligible children who receive PDC. Only one fifth of Medicaid-eligible children utilized PDC [7]. The office presented lists of reasons that led to the low proportion of PDC among Medicaid-eligible children. The reasons include Medicaid low imbursements, low number of dentists who participate in Medicaid, difficulty in obtaining PDC, unreasonable rejections of payments, slow payments, preapproval requirements for dental services, and dentists not willing to treat young children. Medicaid families are also blamed for their under utilization of PDC because they do not see PDC as a high priority practice, plus they like to receive an immediate treatment than waiting for dental appointment or make transportation arrangements [7].

## 6. Contributing Factors

Contributing factors of preventive dental utilization at micro, meso, and macro levels are briefly discussed below.

### 6.1. Micro Level (Individuals and Families)

 The lack of dental coverage is the first influencing factor of accessing, utilizing, and receiving PDC among Medicaid-eligible beneficiaries [32]. Dentists' skills, attitudes, and behaviors have huge impacts on accessing PDC. Some dentists are not willing to treat very young children or participate in Medicaid due mainly to low reimbursements and payments issues which would negatively affect their income [7]. The relationship between Medicaid families and dental care providers may lead to underutilizing PDC. Some Medicaid families experienced disrespectful treatments from dental staff, discrimination, and long waiting time [19].

Knowledge of Medicaid families, attitudes, and behaviors largely affect the utilization of PDC. Families may not place having regular PDC as high priority, and they may lack the knowledge of how important is PDC [7]. Due to the little awareness of the necessity of PDC and as long as the child does not require any immediate pain relief, Medicaid families may not like to book or wait for a PDV and may think that it is worthless to make a traveling effort for only PDV [7]. In addition, family demographic factors such as ability to speak English can affect PDC utilization [37].

 The use of PDC is also predicted by the child's age at his/her first PDV, and it has been proven that the younger the child's age at the first PDV, the more likely he/she utilizes more PDV in the future [5]. Up-to-date well-child care visits can promote PDC among Medicaid-enrolled children because families are influenced and encouraged to seek PDC in the medical office [37].

### 6.2. Meso Level (Community and Organization)

 Communities play huge role in improving PDC access through outreach Medicaid-eligible beneficiaries and educate them about the benefits they generate by maintaining their PDC. Convenient transportations and quality of general education provided are also community level factors that could lead to minimize dental health disparities [20]. The number of dentists and the type of dental provider who participate in Medicaid are important influencing factors in accessing and utilizing PDC [5, 19]. Since the majority of dentists 84% do not participate in government-sponsored programs such as Medicaid [20], there is a huge need to increase their participation in Medicaid and increase dentist-to-population ratio.

 Referral partnership between community resources and medical and/or dental offices has great impact on connecting Medicaid-eligible children to receive appropriate PDC. In addition, the collaboration between medical and dental offices should be promoted in order to fully utilize PDC available [37]. It is important for communities to step up their education for Medicaid-eligible beneficiaries on how to enroll and access the health and dental care systems [27].

### 6.3. Macro Level (Government)

 Medicaid itself sounds very promising and convincing in decreasing PDC disparities among the U.S. children as a whole. Unfortunately, there are many policies and administrative details within Medicaid that have negatively affected the access to PDC and decreased the utilization level among Medicaid-eligible children. Medicaid contributing factors include amount of reimbursements, explanations and communication for denied dental claims, claims processing time, paperwork, and complexity of filling dental claims [7, 23]. Furthermore, the child's age at which Medicaid dental benefits end can be a limitation of utilizing PDC as it is the case in North Carolina [37]. Lastly, assigning dental case mangers for Medicaid beneficiaries may reduce the number of broken appointments [24]. Mangers can identify potential barriers, solve them, and educate clients about PDC [35].

## 7. Outcome

Issues related to effectiveness, efficacy, and equity will be presented in the next part. Outcome evaluation is the sixth section before the conclusion of this paper.

### 7.1. Effectiveness

 The variety between different states in the way they implement and interpret Medicaid has great effect on the way beneficiaries appreciate Medicaid or not. While Oregon State and Washington State Medicaid-enrolled beneficiaries have a positive feedback about Medicaid, beneficiaries from North Carolina, Alabama, and Connecticut are not happy with the current Medicaid program. For example, through implementing the Access to the Baby and Childhood Dentistry (ABCD) that targets Medicaid-enrolled children in Washington State, ABCD is found to be positively effective in providing at least one PDV for high number of Medicaid children [34]. ABCD dentists receive clinical and behavior trainings, and their reimbursements is expanded; in addition, beneficiaries are reached through ABCD and are referred to ABCD dentists by pediatricians and family physicians [34].

 On the other side, Medicaid children in Connecticut complain about access to not only PDC, but to most dental care services [28]. The case is not much different in Georgia and Alabama where the majority of Medicaid children are less likely to receive PDC [30]. In general, only few Medicaid-eligible children received at least single PDC [7]. Among those who received PDC, only few receive dental treatments [29].

### 7.2. Efficiency

 The argument that Medicaid saves money through PDC is not an easy argument. The total dental dollar amounts reimbursed for Medicaid-enrolled children in 2002 range from $19,113 to $72,614 [32]. Seventy-nine percent of Medicaid-eligible children receive PDC and have the least dental expenses. On the other hand, Medicaid children with high dental expenses and more dental problems consume 64% of Medicaid money, and they only represent 9% of the total Medicaid-enrolled children [32].

 Medicaid children who receive PDC at early stage have fewer dental-related expenses than those who start dental care at a later time. The average dental cost for each Medicaid child who receives early PDC is only $263 during 5 years [5]. Although the children who receive PDC usually have little dental expenses, the dollar amount saved is consumed by children with more dental problems and high expenses. Medicaid needs to increase the number of children who receive PDC and decrease the percentage of children with high dental expenses in the same time.

 Medicaid can reach the objective of saving money from PDC through better outreach, get children see dentists as early as possible, increase the number of dentists who participate in Medicaid, and provide easy PDC access to Medicaid-eligible children. Overall, Medicaid needs to increase the amount of money assigned for PDC reimbursements as it is shown to be the most influencing factor for dentists to participate in Medicaid. Increasing the amount of reimbursements can help Medicaid-enrolled children in having a convenient access to PDC and better utilization of the services.

### 7.3. Equity

 Equity is about equal access to PDC. Medicaid program plays huge role in reducing oral health disparities among U.S. children. Children with either private or public dental coverage are more likely to access and utilize more PDC than their peers who lack dental coverage. Unfortunately, the shortage in the number of dentists who participate in Medicaid and other factors related to Medicaid-eligible beneficiaries have huge impacts on not reaching the goal of eliminating access disparities to PDC [5, 7, 19, 20, 23, 32, 37].

## 8. Conclusion

Although dental disparities among the U.S. children are decreased after Medicaid was enacted and expanded, oral health disparities in PDC still present among the U.S. children. Despite the recent increasing number of children enrolled in Medicaid, accessing and utilizing PDC is the real challenge that faces Medicaid. Adapting different plans and strategies from different states which witness success within their Medicaid programs is recommended.
